# Impact of highly deleterious non-synonymous polymorphisms on GRIN2A protein’s structure and function

**DOI:** 10.1371/journal.pone.0286917

**Published:** 2023-06-15

**Authors:** Ishtiaque Ahammad, Tabassum Binte Jamal, Arittra Bhattacharjee, Zeshan Mahmud Chowdhury, Suparna Rahman, Md Rakibul Hassan, Mohammad Uzzal Hossain, Keshob Chandra Das, Chaman Ara Keya, Md Salimullah

**Affiliations:** 1 Bioinformatics Division, National Institute of Biotechnology, Ganakbari, Ashulia, Savar, Dhaka, Bangladesh; 2 Department of Biochemistry and Microbiology, North South University, Bashundhara, Dhaka, Bangladesh; 3 Molecular Biotechnology Division, National Institute of Biotechnology, Ganakbari, Ashulia, Savar, Dhaka, Bangladesh; University of Michigan, UNITED STATES

## Abstract

*GRIN2A* is a gene that encodes NMDA receptors found in the central nervous system and plays a pivotal role in excitatory synaptic transmission, plasticity and excitotoxicity in the mammalian central nervous system. Changes in this gene have been associated with a spectrum of neurodevelopmental disorders such as epilepsy. Previous studies on *GRIN2A* suggest that non-synonymous single nucleotide polymorphisms (nsSNPs) can alter the protein’s structure and function. To gain a better understanding of the impact of potentially deleterious variants of *GRIN2A*, a range of bioinformatics tools were employed in this study. Out of 1320 nsSNPs retrieved from the NCBI database, initially 16 were predicted as deleterious by 9 tools. Further assessment of their domain association, conservation profile, homology models, interatomic interaction, and Molecular Dynamic Simulation revealed that the variant I463S is likely to be the most deleterious for the structure and function of the protein. Despite the limitations of computational algorithms, our analyses have provided insights that can be a valuable resource for further *in vitro* and *in vivo* research on *GRIN2A-*associated diseases.

## Introduction

The goal of human genetics research is to comprehend how frequent genetic variations affect the probability of getting common illnesses [1]. Single Nucleotide Polymorphisms (SNPs) which refer to single base substitution in the alleles are such common sources of variance in the genome that can significantly influence an individual’s physical characteristics and their chance of developing certain diseases [2]. SNPs in the coding regions of genes (cSNPs) or regulatory regions are more likely to cause functional differences than SNPs elsewhere. About 90% of the variation in the human genome is due to SNPs, making them the most common type of genetic alteration [3].

Various genetic tools and related genomics have been used for the large-scale extraction of SNPs over the years. Over 10 million SNPs have been found, covering the most common types of genetic variations [4]. Since the human genome covers a vast number of genetic polymorphisms, it is necessary to conduct extensive research to understand the significance of each one and how they may contribute to disease susceptibility and personalized drug development [1]. To scale back the amount of effort required, various computational methods have been developed to identify potential variants before testing them in laboratory conditions [5]. In this circumstance, *in silico* approach is an efficient way to determine which SNPs are harmful and which are not, using particular processes [6]. Besides, integrative analysis of various algorithms improves the accuracy of the predicted effects of particular mutations. Moreover, cutting-edge approaches, such as molecular dynamics simulation allow for precise assessment of changes in protein properties, including structure, chemical properties, and interactions, within a simulated environment [7–9].

SNPs that occur in the coding region can be either synonymous, meaning they do not cause any change in the amino acid sequence, or non-synonymous (nsSNPS), meaning the amino acid sequence is altered [10]. It is usually assumed that synonymous SNPs are harmless, as the protein’s primary sequence is not changed [11]. On the other hand, nsSNPs may subsequently affect protein structure and protein-protein interactions and exert possible functional effects. However, not all nsSNPs that cause structural and functional changes are potentially harmful. Some nsSNPs affect the structure of a protein, while others create functional consequences. Moreover, some nsSNPs may be linked to disease, while others are considered neutral and do not have any association with disease [12–15]. Therefore, it is crucial to differentiate between harmful and neutral nsSNPs. In this work, we aimed to determine the most harmful nsSNPs within the *GRIN2A* gene by evaluating their impact on the structure and function of the GluN2A protein.

*GRIN2A* gene encodes for the GluN2A subunit of the N- methyl-D-aspartate (NMDA) glutamatergic receptor that is expressed throughout the brain and is important in the function of all neuron types. This receptor is activated through simultaneous binding by Glu and glycine and is formed by combinations of NR1, NR2, and NR3 subunits, with NR1-NR2 heterodimers forming the basic functional structure. The NR1 subunit has glycine binding sites, while the NR2 subunit has glutamate binding sites. NR3 subunits are regulatory subunits that decrease ionic currents generated by NR1/NR2 heteromers and are probably involved in activating silent NMDA-alone synapses. NMDA receptors are essential for synaptic transmission, learning, and memory as they function as ion channels that allow positively charged ions to flow through the neuron’s membrane [16]. Any mutations in *GRIN2A* gene weaken ion flow through the receptor, resulting in abnormal neuron function, epilepsy, and related developmental differences [17,18].

*GRIN2A*-related speech disorders and epilepsy affects individuals from a young age. All affected ones display some degree of speech disorder, with severe cases including dysarthria and speech dyspraxia, and both receptive and expressive language delay or regression. In addition to speech disorders, about 90% of affected individuals also experience epilepsy, with seizure onset typically occurring between the ages of three and six. The seizures associated with *GRIN2A* mutations can take various forms, including seizures with an aura of perioral paresthesia, focal or focal motor seizures, and atypical absence seizures. Different epilepsy syndromes including Landau-Kleffner syndrome, epileptic encephalopathy, childhood epilepsy, autosomal dominant rolandic epilepsy and infantile-onset epileptic encephalopathy have been linked to *GRIN2A* mutations [19]. However, the proportion of the abnormalities caused by *the GRIN2A* pathogenic variants is yet to be identified. The abundance of SNPs present in the *GRIN2A* makes laboratory experimentations challenging, seeking to investigate the functional effects of these variations, as such experiments can be both expensive and time-consuming. Therefore a computational screening of SNPs to minimize the number of potential pathogenic ones is a must before experimental mutation analysis. Considering this fact, in the current study we aimed to predict the consequences of the most damaging nsSNPs that occur in the *GRIN2A* coding region as reported in the dbSNP database (https://www.ncbi.nlm.nih.gov/snp/).

## Materials and methods

**Fig 1:**
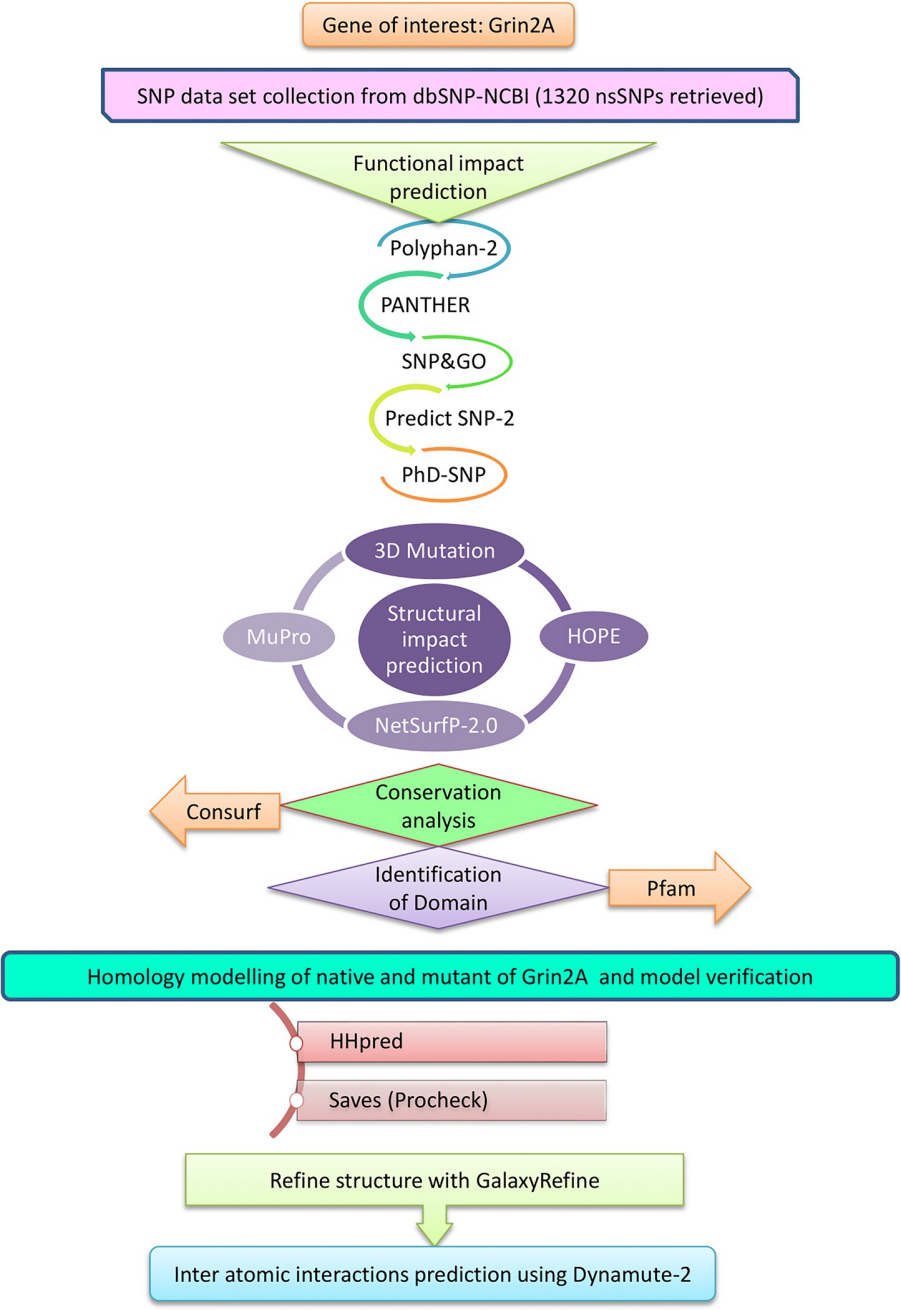
Schematic representation of the workflow of the study. The overall process can be summarized as a series of steps that involve filtering and identifying the most damaging nsSNPs of GRIN2A, followed by a subsequent in-depth analysis of one particular nsSNP that proved to be the most deleterious.

The complete workflow employed in this study is outlined in **Fig 1.**

### Retrieval of SNPs

Single Nucleotide Polymorphism (SNP) variants recorded for the human *GRIN2A* gene were retrieved from NCBI dbSNP [20]. Only missense variants were selected for our study. The representative protein sequence of this gene was obtained from UniProt (https://www.uniprot.org/) (UniProt ID: Q12879) [21].

### Functional evaluation of the nsSNPs

Five different tools were used to predict the impact of nsSNPs on the *GRIN2A*. These tools include PolyPhen-2, PANTHER, SNPs & GO, PhD-SNP, and PredictSNP2.

The functional and structural effects of certain missense variants were analyzed using several online tools. At first, the variants were submitted to the PolyPhen-2 (http://genetics.bwh.harvard.edu/pph2/) server, which is used to predict the effects of human nsSNPs on the function of protein molecules. This server, which is based on sequence homology, can help identify potentially damaging missense mutations and analyze the potential effects of an amino acid substitution on the structure and function of a human protein [22]. The rsIDs (Example: chr16:10274423 A/G) retrieved from the dbSNP database were used as input queries.

PANTHER (http://pantherdb.org/tools/csnpScoreForm.jsp) is a software that is used to predict non-synonymous genetic variants that may be related to human disease. This tool uses position- specific evolutionary conservation (PSEC) scores to make these predictions [23].

SNPs&GO (https://snps.biofold.org/snps-and-go/pages/method.html) is an online server that combines data from protein sequences, evolutionary information, and functions encoded in Gene Ontology terms to predict the effects of SNPs. This server is considered to be more accurate than other similar methods available [24].

PHD-SNP (https://snps.biofold.org/phd-snp/phd-snp.html) is a tool for predicting the effects of deleterious single nucleotide polymorphisms in humans, which is based on a Support Vector Machine (SVM) classifier. This tool can take the protein sequence, position of the mutation, or the mutated residue as input query. PMut (http://mmb.irbbarcelona.org/PMut/) is another web- based tool for predicting the effects of nsSNPs on protein function. A score greater than 0.5 obtained from this tool indicates that the nsSNPs have a damaging impact on protein function [25].

PredictSNP2 (https://loschmidt.chemi.muni.cz/predictsnp2/) is an online interface that provides easy access to predictions from five different tools and their consensus scores in a user-friendly format. It is tailored to the specific features of various categories of variations. The predictions are also accompanied by annotations from relevant databases to enable a comprehensive evaluation of variants [26].

### Structural impact prediction

Four different structural impact prediction tools (NetSurfP-2.0, MUpro, Mutation3D, and HOPE) were used to find out the effect of SNPs on the protein structure.

NetSurfP-2.0 (https://services.healthtech.dtu.dk/service.php?NetSurfP-2.0) is a relatively new sequence-based tool that can accurately predict important local structural features of proteins with fast computation time. This server uses a neural network architecture that includes convolutional and long short-term memory layers, trained on previously solved protein structures [27]. The FASTA sequence of the GRIN2A protein was submitted to NetSurfP, and the output of the server revealed the buried and exposed regions in the protein structure [28].

With a prediction accuracy of 84%, MUpro (https://www.ics.uci.edu/~baldig/mutation.html) utilizes both Support Vector Machines and Neural Networks to calculate the impact of point mutations on protein stability. Similarly to NetSurfP2.0, it requires the FASTA format of the protein sequence as input [29].

Mutation3D (http://mutation3d.org) is a web server that aims to identify driver genes in cancer by detecting clusters of amino acid substitutions within tertiary protein structures [30]. The 3D structure of GRIN2A protein can be predicted and evaluated using mutation3D. To find out whether a mutation falls inside a domain, Mutation3D can be utilized.

Have (y) Our Protein Explained (HOPE) server (https://www3.cmbi.umcn.nl/hope/about/) uses a comprehensive approach to analyze a specific mutant protein. It gathers data from various sources, including 3D coordinate calculations of the protein. Before conducting analysis, this server requires the native protein sequence and information on the specific location and type of mutation to be studied [31].

### Conservation profile analysis

To unravel the evolutionarily conserved sites of the human GRIN2A protein, the Consurf web server (http://consurf.tau.ac.il) was used. This server, which has been in operation for over 15 years, uses evolutionary patterns of amino/nucleic acids to identify regions that are critical for structure and/or function [32].

### Identification of domain

For domain identification the Pfam server (http://pfam.xfam.org/) was employed. This is a widely used tool for analyzing protein function that features a collection of curated protein families, each defined by two alignments and a profile hidden Markov model (HMM). The profile HMMs, generated by aligning a set of family-representative sequences, are statistical models used to search for similarities among proteins. The Pfam website provides several methods to access its database content, including graphical representations and interactive data access [33].

### Homology modeling and verification of native and mutant forms of GRIN2A protein

HHpred (https://toolkit.tuebingen.mpg.de/tools/hhpred) is a web-based tool for detecting remote protein homology and predicting structures that extracts homology information from HH-suite (open-source software for searching sensitive protein sequences). It is one of the first servers to effectuate pairwise comparison of profile hidden Markov models (HMMs). It accepts a single query sequence and multiple alignments as input depending on the user’s preference [34]. The protein FASTA sequences were submitted as input to generate the homology model of the protein. Later, the structure quality was exposed to verification by PROCHECK. The PROCHECK tool (https://servicesn.mbi.ucla.edu/PROCHECK/) evaluates the structural integrity of a protein by looking into the geometry of individual residues and the overall structure of the protein [35]. The model structure was further verified using the SAVES web server, and the quality of the structure was confirmed by analyzing the Ramachandran plot. Usually, a protein structure is considered good if more than 90% residues are in the favored region of Ramachandran plot. These techniques play a fundamental role in understanding the 3D models of proteins.

### Refinement of the 3D structure of GRIN2A

To improve the accuracy of the 3D model for the GRIN2A protein, GalaxyRefine (https://galaxy.seoklab.org/cgibin/submit.cgi?type=REFINE) server was employed. This is a web-based tool for predicting and refining protein structures along with other related methods. It processes protein structure by performing repeated perturbation and structural relaxation by molecular dynamics simulation. The mechanism starts with the reconstruction of side chains and continues with side-chain repacking and overall structure relaxation [36].

### Interatomic interactions prediction

The nsSNPs selected from the upstream analysis were evaluated using the DynaMut2 server. DynaMut2 (https://biosig.lab.uq.edu.au/dynamut2/) is an all-inclusive tool for analyzing protein motion and flexibility. It integrates optimized graph-based signatures with normal mode parameters to estimate the impact of point mutations on protein stability. Not only that but, it also predicts the effect of missense variations on protein stability and dynamics which is critical for understanding the link between protein structure and function and their role in associated diseases [37].

### Molecular dynamic simulation

The wild-type GRIN2A and selected mutant protein models were subjected to a 100 ns molecular dynamics simulation using the GROMACS (version 2020.6) simulation software [38]. The force field utilized in the simulation was GROMOS96 43a1. A waterbox with edges 0.5 nm from the protein surface was constructed using the spc216 water model. Appropriate ions were used to neutralize the systems. A 100 ns molecular dynamic simulation was performed utilizing periodic boundary conditions following energy minimization, isothermal-isochoric (NVT) equilibration, and isobaric (NPT) equilibration of the system. After the simulation was complete, the root mean square deviation (RMSD), root mean square fluctuation (RMSF), the radius of gyration (Rg), and solvent accessible surface area (SASA) analyses were performed using the RMSD, RMSF, Rg, and SASA modules integrated into the GROMACS software. The plots for each of these studies were produced using the ggplot2 package in RStudio.

## Results

### SNP retrieval

Single nucleotide Polymorphism (SNP) of the human *GRIN2A* gene (Uniprot ID Q12879) was retrieved from the NCBI database (https://www.https://www.ncbi.nlm.nih.gov/snp/). Out of the 179,447 SNPs covered, 1,320 were missense (nsSNPs).

### Assessment of the functional effect of *GRIN2A* nsSNPs

To evaluate the functional effects of nsSNPs on *GRIN2A* gene product, five tools were used: PolyPhen 2, PANTHER, SNPs&GO, PhD-SNP, and Predict SNP2. Out of the total 1320 nsSNPs, PolyPhen 2 server predicted 832 nsSNPs to be functionally damaging with 490 labeled as "probably damaging" and 275 as "possibly damaging." To further investigate these SNPs, additional analysis was conducted using SNPs&GO, PANTHER, PhD-SNP, and Predict-SNP2. The number of detrimental ones predicted by Sthe NPs&GO server was 154 while PANTHER, PhD-SNP, and Predict-SNP2 predicted 141, 451, and 692 nsSNPs as disease-associated, respectively.

Based on the outputs, 16 most highly deleterious nsSNPs were selected that were labeled as damaging, possibly/probably damaging, or deleterious by these tools. These 16 functionally significant nsSNPs were further taken into consideration for the next stage of filtering. The results from PolyPhen 2, PANTHER, SNPs&GO, PhD-SNP, and Predict SNP2 are presented in **Table 1.**

**Table 1:** Identification of deleterious nsSNPs using PHD-SNP, SNPs&GO, PANTHER, Polyphen-2, and Predict SNP2.

Amino Acid Mutation	PhD-SNP	SNPs&GO	PANTHER	Polyphen-2	Predict SNP2
R52Q	Disease	Disease	Disease	Probably damaging	Deleterious
G9C	Disease	Disease	Disease	Probably damaging	Deleterious
G97S	Disease	Disease	Disease	Probably damaging	Deleterious
Q146P	Disease	Disease	Disease	Probably damaging	Deleterious
L361Q	Disease	Disease	Disease	Probably damaging	Deleterious
G376S	Disease	Disease	Disease	Probably damaging	Deleterious
D462E	Disease	Disease	Disease	Probably damaging	Deleterious
I463S	Disease	Disease	Disease	Probably damaging	Deleterious
V471M	Disease	Disease	Disease	Probably damaging	Deleterious
M507T	Disease	Disease	Disease	Probably damaging	Deleterious
P527S	Disease	Disease	Disease	Probably damaging	Deleterious
L642M	Disease	Disease	Disease	Probably damaging	Deleterious
A643T	Disease	Disease	Disease	Probably damaging	Deleterious
S644G	Disease	Disease	Disease	Probably damaging	Deleterious
E656K	Disease	Disease	Disease	Probably damaging	Deleterious
A778D	Disease	Disease	Disease	Probably damaging	Deleterious

### Structural impact prediction of *GRIN2A* nsSNPs

Sixteen nsSNPs that had been predicted to be damaging by 5 different tools then underwent structural impact analysis. For this purpose, four servers were employed namely, Mutation3D, NetSurfP-2.0, MUpro, and HOPE.

### Three-dimensional visualization of *GRIN2A* mutations

**Fig 2:**
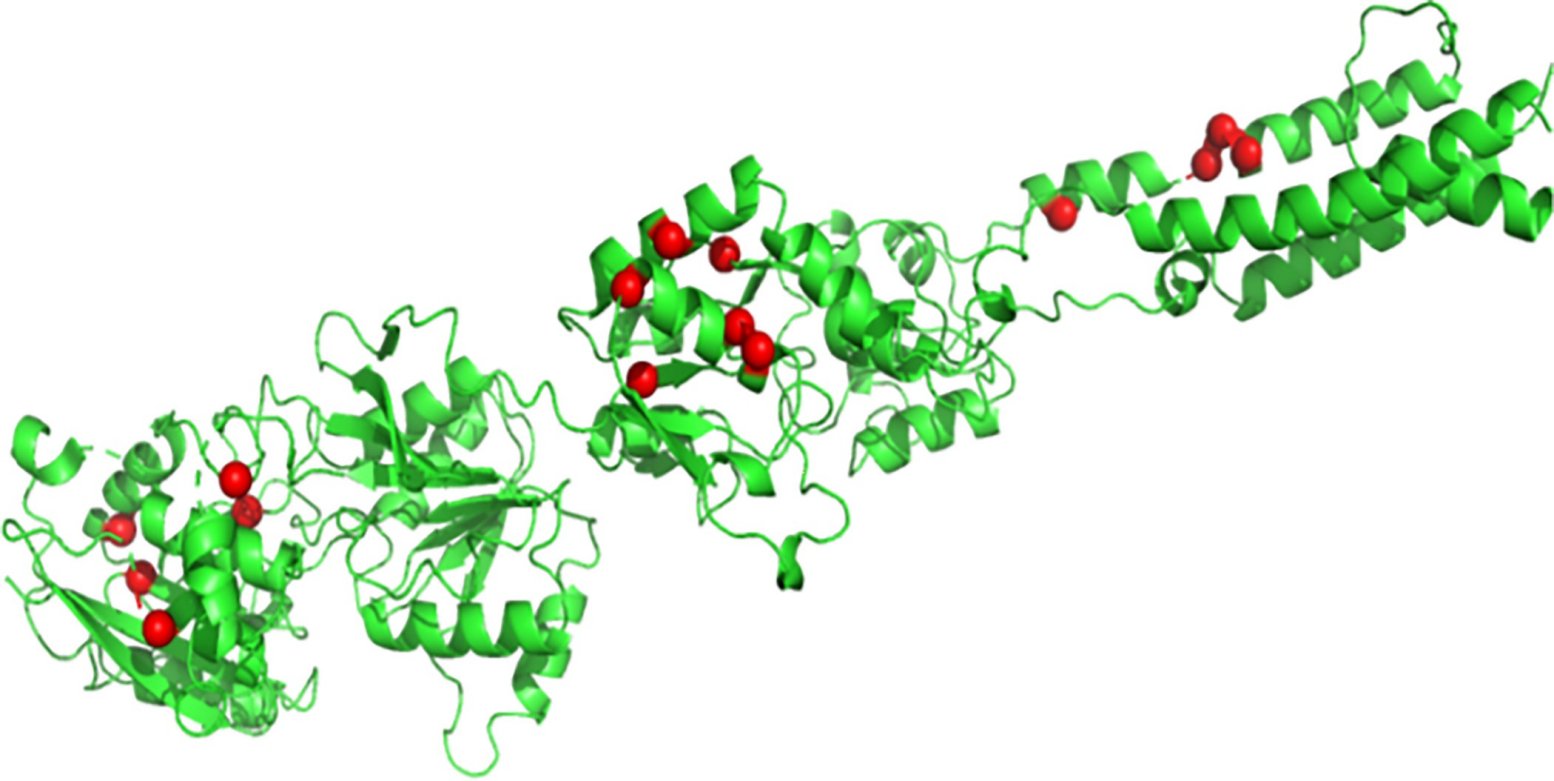
Locations of 16 deleterious nsSNPs of GRIN2A as revealed by the Mutation3D server.

For visualizing the locations of 16 nsSNPs of *GRIN2A* on the protein, outputs from the Mutation 3D server were loaded onto the PyMol software. The result was a three-dimensional representation of the human GRIN2A protein (**Fig 2**), with the mutated residues highlighted in red.

### Surface accessibility of native and mutant proteins

Native and mutant protein accessibility and stability for the 16 variants were assessed by NetsurfP-2.0. NetSurfP-2.0 predicts the degree to which a residue is buried or exposed within the protein structure, as a percentage. These predictions were substantiated using multiple distinct test datasets. Out of 16 deleterious nsSNPs, 3 (R52Q, A643T, E656K) were found to be exposed in the wild type, while the remaining 13 (G97C, G97S, Q146P, L361Q, G376S, D462E, I463S, V471M, M507T, P527S, L642M, S644G, A778D) were buried. On the other hand, 2 mutants (R52Q, E656K) remained exposed while the rest of the 14 (G97C, G97S, Q146P, L361Q, G376S, D462E, I463S, V471M, M507T, P527S, L642M, A643T, S644G, A778D) were buried (**Table 2**).

**Table 2:** Surface accessibility of native and mutant proteins.

Amino Acid residue	Wild Type				Mutant type			
	Relative surface Accessi bility (RSA)	RSA in (%)	Absolute surface Accessibility (ASA) Å	Pdisorder	Relative surface Accessibility (RSA)	RSA in (%)	Absolute surface Accessibility (ASA) Å	Pdisorder
R52Q	exposed	4%	101	0%	exposed	39%	70	0%
G97C	buried	1%	1	0%	buried	1%	1	0%
G97S	buried	1%	1	0%	buried	1%	1	0%
Q146P	buried	4%	7	0%	buried	3%	4	0%
L361Q	buried	2%	4	0%	buried	5%	9	0%
G376S	buried	7%	5	0%	buried	8%	9	0%
D462E	buried	24%	34	0%	buried	26%	45	0%
I463S	buried	1%	1	0%	buried	1%	1	0%
V471M	buried	8%	12	0%	buried	9%	17	0%
M507T	buried	1%	2	0%	buried	1%	2	0%
P527S	buried	13%	18	0%	buried	13%	15	0%
L642M	buried	16%	29	0%	buried	12%	24	2%
A643T	exposed	28%	31	3%	buried	26%	36	2%
S644G	buried	22%	26	4%	buried	19%	15	3%
E656K	exposed	60%	105	7%	exposed	56%	116	7%
A778D	buried	3%	4	0%	buried	12%	18	0%

### Structural impact of the SNPs on *GRIN2A* gene product

Out of 16 nsSNPs, the MUpro server predicted that 14 nsSNPs would have a decreasing effect on protein stability. The Project HOPE server was used to investigate the effect of mutations on various aspects of protein structure and function such as physico-chemical properties, hydrophobicity, intermolecular interactions, and structural and functional changes. In accordance with the results, 10 mutant residues (G97C, G97S, L361Q, G376S, D462E, V471M, L642M, A643T, E656K, and A778D) were larger than the wild type, while 6 mutant residues (R52Q, Q146P, I463S, M507T, P527S, and S644G) were smaller than the wild type. Out of the 16 nsSNPs analyzed, 14 were predicted to have a probable damaging effect, one as a possibly damaging effect and one as a damaging effect on the protein structure according to the HOPE server.

### Identification of conserved sequence in *GRIN2A*

To further examine the potential impact of the shortlisted 16 nsSNPs, the ConSurf web tool was used to calculate the evolutionary conservation of amino acid residues in the GRIN2A protein. The results were presented as a structural representation of the protein sequence, with the putative structural and functional residues highlighted.

**Fig 3:**
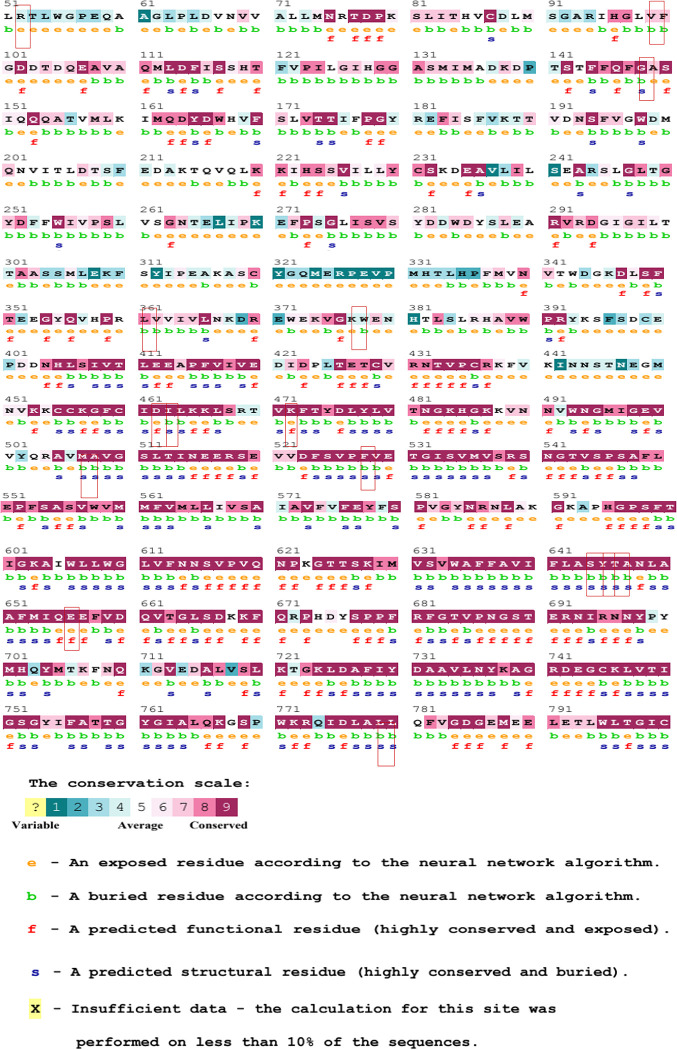
Evolutionary conservation profile of amino acid residues as predicted by ConSurf. For the identification of the most deleterious nsSNPs, ConSurf, MUpro, and HOPE outputs were analyzed and scrutinized. Finally, 9 out of the 16 mutants were considered the most impactful for the structure of the GRIN2A protein and selected for further analysis.

According to ConSurf output, 12 of the 16 nsSNPs (Q146P, G376S, D462E, I463S, V471M, M507T, P527S, L642M, A643T, S644G, E656K, and A778D) were found to be highly conserved residues with a conservation score of 9. Three variants (G97C, G97S, and L361Q) were predicted as moderately conserved (conservation score of 8) while only one variant (R52Q) was predicted as a variable with a conservation score of 3. Variants located in these conserved regions are considered to be highly damaging to the protein, as referred to as those located in non-conserved sites. The deleterious predictions for each SNP by ConSurf are summarized in **Fig 3**. After careful analysis, we identified 9 out of 16 mutants as having the most significant impact on the structure of the GRIN2A protein. We subsequently selected specific nsSNPs (listed in **Table 3**) from the GRIN2A protein for further investigation.

**Table 3:** Prediction of High-Risk nsSNPs of *GRIN2A* Gene according to Consurf, MUPro, HOPE.

Mutation	Consurf	MUPro	HOPE
Q146P	CONSERVED	Decrease	Probably Damaging
I463S	CONSERVED	Decrease	Probably Damaging
V471M	CONSERVED	Decrease	Probably Damaging
M507T	CONSERVED	Decrease	Probably Damaging
P527S	CONSERVED	Decrease	Probably Damaging
A643T	CONSERVED	Decrease	Probably Damaging
S644G	CONSERVED	Decrease	Probably Damaging
E656K	CONSERVED	Decrease	Probably Damaging
A778D	CONSERVED	Decrease	Probably Damaging

### Identification of domain

Three domains, namely- ANF receptor, Lig chan-Glu, and Lig chan were identified within the GRIN2A protein using the Pfam server. The ANF receptor domain contained 1 nsSNP (Q146P), while the Lig chan-Glu domain had 4 nsSNPs (I463S, V471M, M507T, P527S) and the Lig chan domain had 4 nsSNPs (A643T, S644G, E656K, A778D).

### Homology modeling and validation of the GRIN2A structure

The homology model of the protein was generated in PDB Format using the HHPred server. The quality of the model was validated by inspecting the Ramachandran plot generated by the PROCHECK web server. The findings indicated that over 90% of the residues of the homology model belonged to the most favored regions for both the wild and mutant version (**S1 Fig**).

### Refinement of the 3D structure

The 3D model of the protein of interest was refined using the GalaxyRefne server. The input for the refinement process was in PDB file format. The server provided information on the 5 best models in terms of RMSD, Clash score, Rama favored, and MolProbity. The model with the lowest MolProbity score and the highest Rama favoured score was selected (**Table 4**).

**Table 4:** Refinement of the *GRIN2A* homology model structure using the GalaxyRefne web server.

SNPs	Selected Model	RMSD	MolProbity	Clash score	Rama favored
Q146P	MODEL 4	0.300	1.606	12.4	99.2
I463S	MODEL 2	0.405	1.961	16.3	96.3
V471M	MODEL 4	0.389	1.973	14.4	95.6
M507T	MODEL 4	0.445	1.846	14.9	97.0
P527S	MODEL 2	0.373	1.888	13.5	96.3
A643T	MODEL 3	0.422	1.857	11.6	96.0
S644G	MODEL 3	0.444	1.767	12.3	97.1
E656K	MODEL 3	0.449	1.784	10.5	96.3
A778D	MODEL 1	0.371	1.749	9.6	96.3

### Interatomic interactions analysis

**Fig 4:**
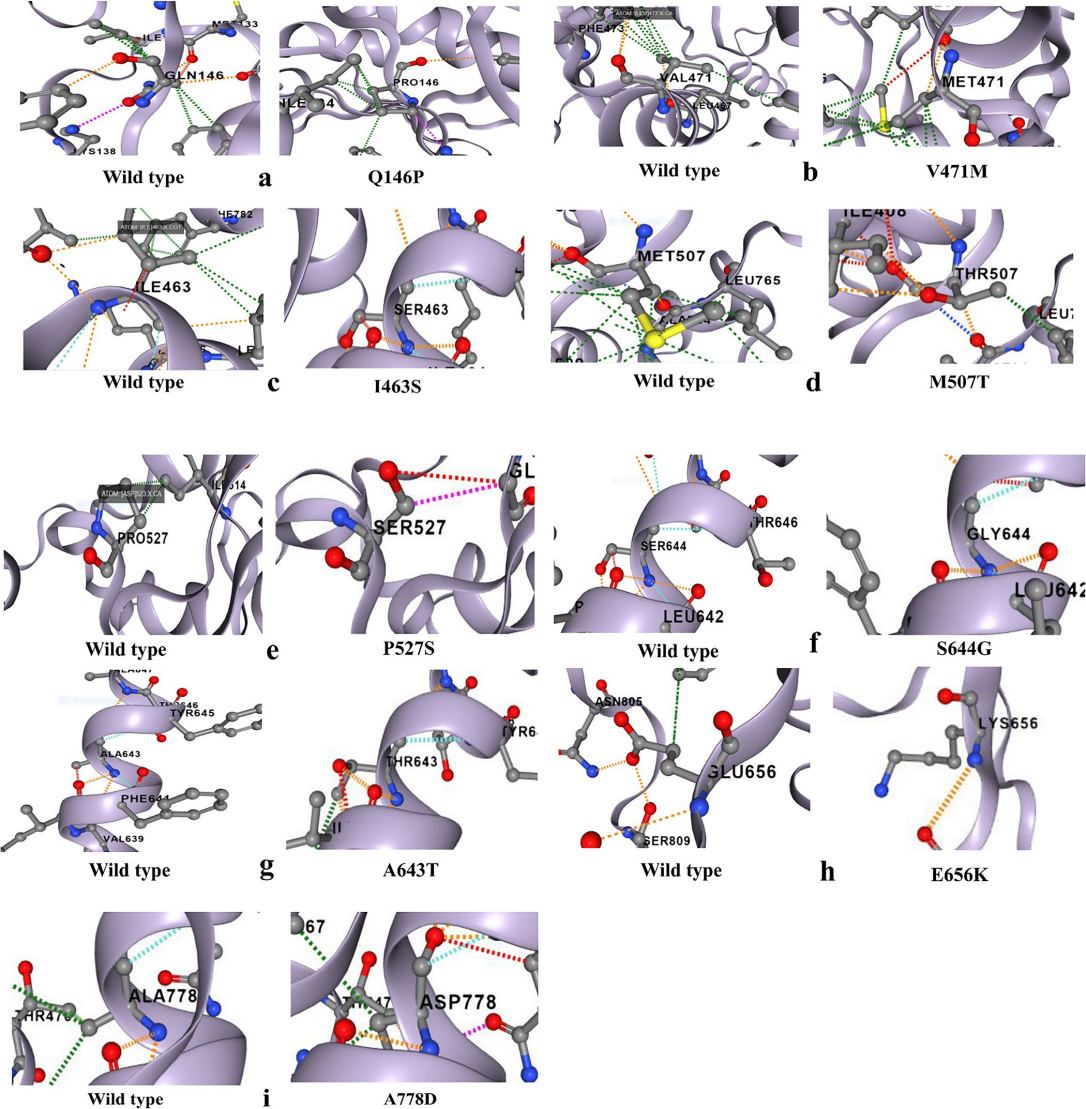
DynaMut2 prediction of inter-atomic interactions of the native DRD2 vs the mutant. Altered interatomic interactions between the wild and the mutant residues with their neighboring atoms have been observed.

The nsSNPs selected so far were analyzed using the DynaMut2 server (**Fig 4, Table 5**). DynaMut2 provides the change in stability (ΔΔG) in terms of kcal/mol. A negative value indicates a destabilizing effect while a positive value indicates a stabilizing effect. The more negative the ΔΔG value the more destabilizing the mutation is for the protein. The analysis identified 8 residues (Q146P, I463S, V471M, M507T, P527S, S644G, E656K, A778D) as destabilizing for the protein structure, while 1 residue (A643T) had a stabilizing effect. The SNP, I463S exhibited the highest destabilizing effect on the protein (-2.61 kcal/mol). Therefore, it was selected for sophisticated molecular dynamics simulation in the next step.

**Table 5:** Predicted changes in stability as a result of mutation.

Mutation	Stability (ΔΔG)	Effect
Q146P	-0.38 kcal/mol	Destabilising
I463S	-2.61 kcal/mol	Destabilising
V471M	-0.61 kcal/mol	Destabilising
M507T	-1.93 kcal/mol	Destabilising
P527S	-1.61 kcal/mol	Destabilising
A643T	0.01 kcal/mol	Stabilising
S644G	-0.55 kcal/mol	Destabilising
E656K	-0.35 kcal/mol	Destabilising
A778D	-0.39 kcal/mol	Destabilising

### Molecular dynamics simulation

**Fig 5:**
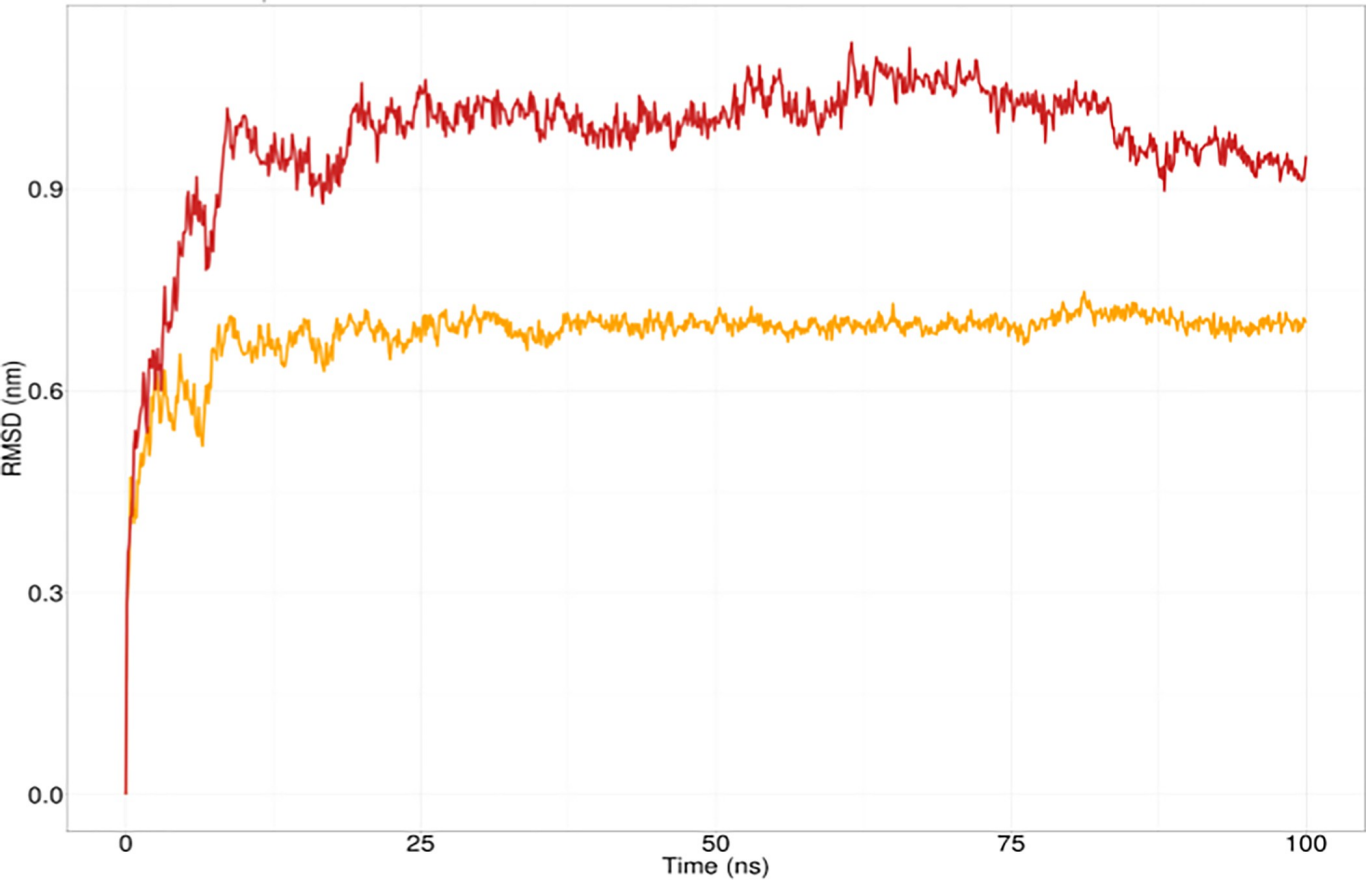
RMSD analysis of wild type GRIN2A (Yellow) and I463S mutant GRIN2A protein (Red).

Root Mean Square Deviation (RMSD) calculation is performed in to evaluate the stability of the systems. A higher RMSD value corresponds to instability in the protein. The RMSD for the wild *GRIN2A* stabilized fairly quickly after 10 ns. Since then, its value remained stable at around 0.7 nm. In contrast, the I463S mutant *GRIN2A* possessed a higher RMSD profile throughout the simulation. Its value ranged above 0.9 nm from 10 ns till the end of the simulation (**Fig 5**).

**Fig 6:**
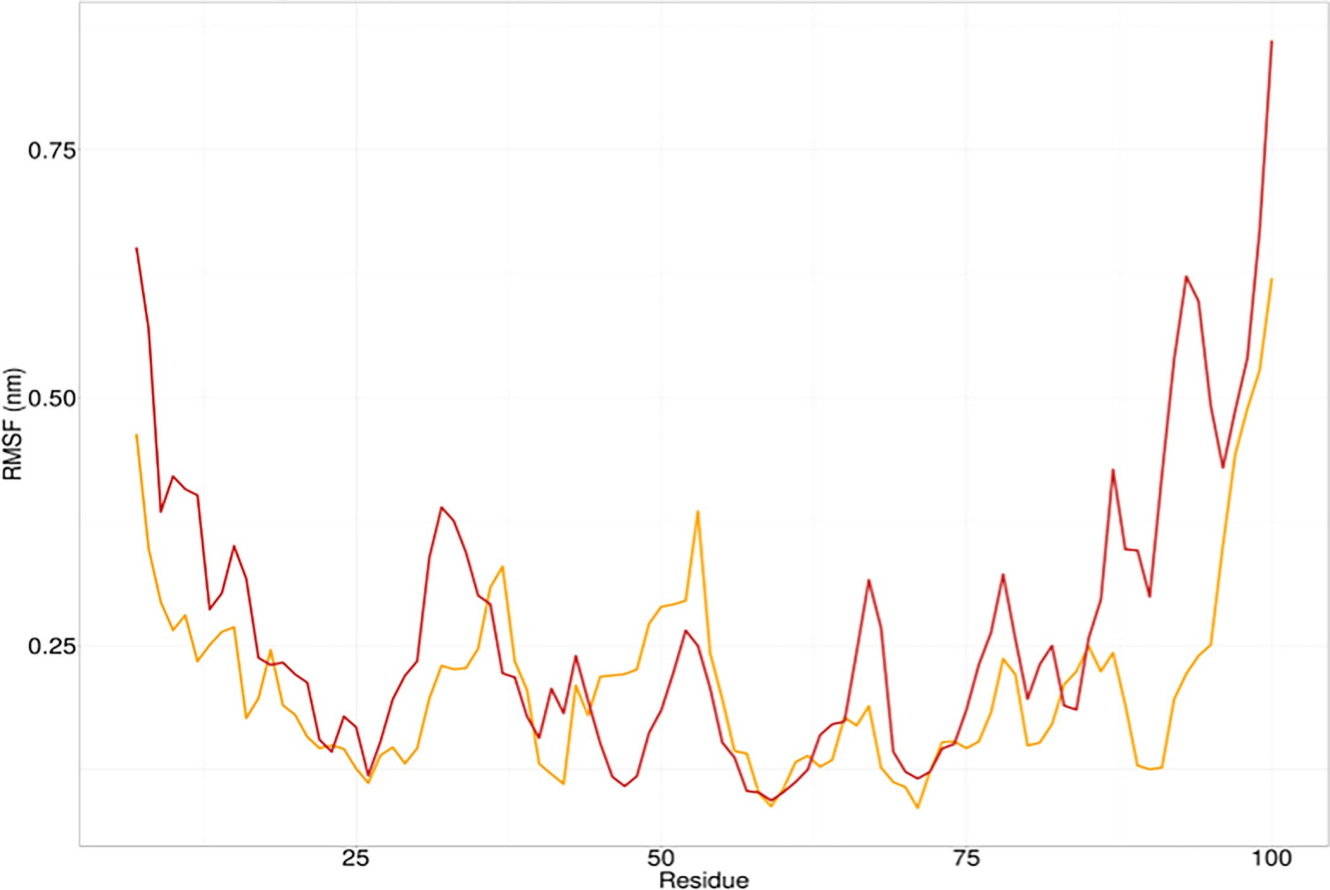
RMSF analysis of wild type GRIN2A (Yellow) and I463S mutant GRIN2A protein (Red).

Room Mean Square Fluctuation (RMSF) is used to determine the regional flexibility of the protein. The higher the RMSF, the higher is the flexibility of a given amino acid position. Except for a few in the middle, almost all residues in the mutant I463S exhibited higher flexibility compared to the wild-type *GRIN2A* residues (**Fig 6**).

**Fig 7:**
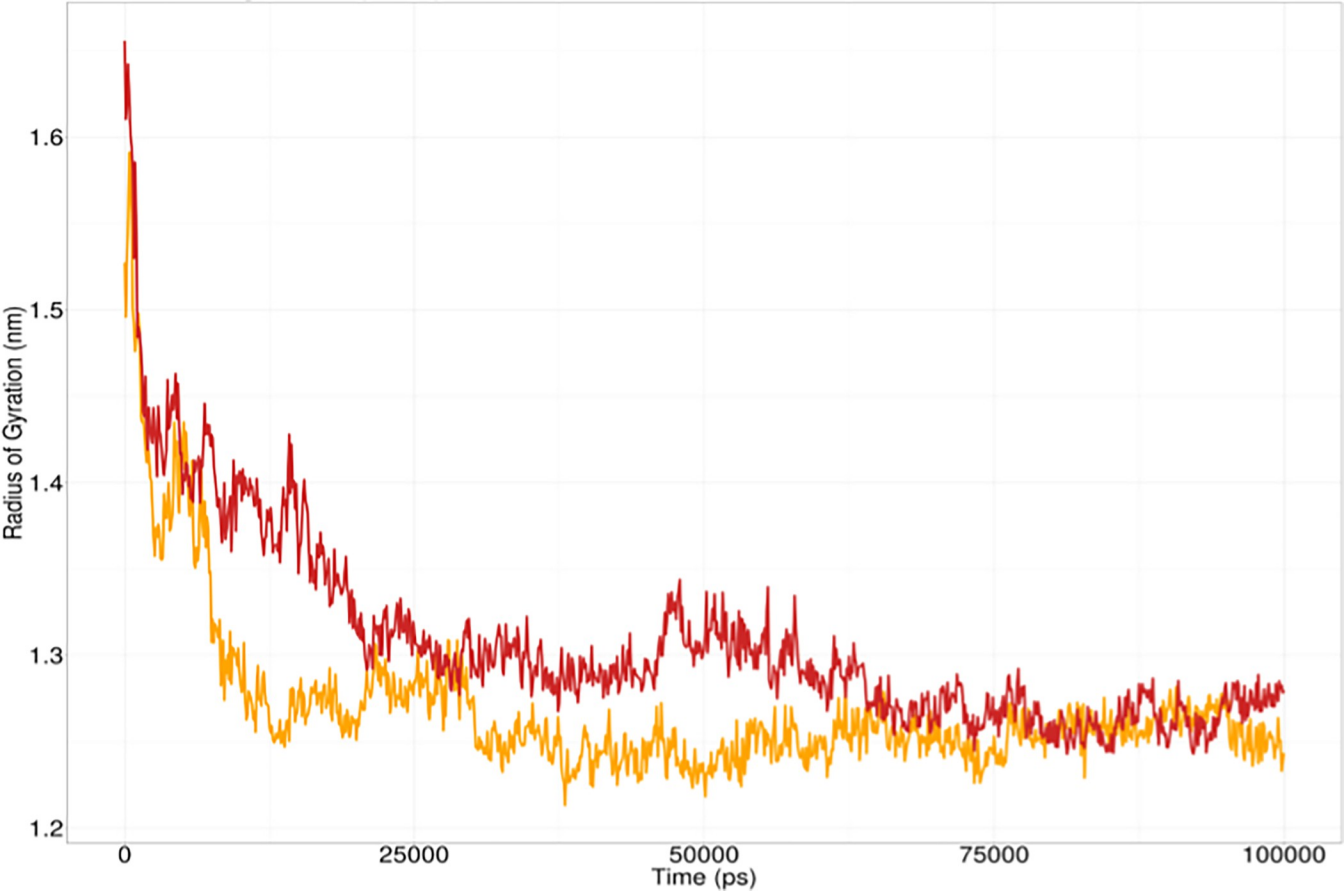
Radius of gyration analysis of wild type GRIN2A (Yellow) and I463S mutant GRIN2A protein (Red).

The radius of gyration is a measure to determine its degree of compactness. A relatively steady value of the radius of gyration means stable folding of a protein. Fluctuation of the radius of gyration implies the unfolding of the protein. The radius of gyration analysis indicated that the wild type *GRIN2A* was relatively more compact than the I463S mutant *GRIN2A* as the former reached a stable value shortly after 35 ns while the latter reached this state after 65 ns (**Fig 7**).

**Fig 8:**
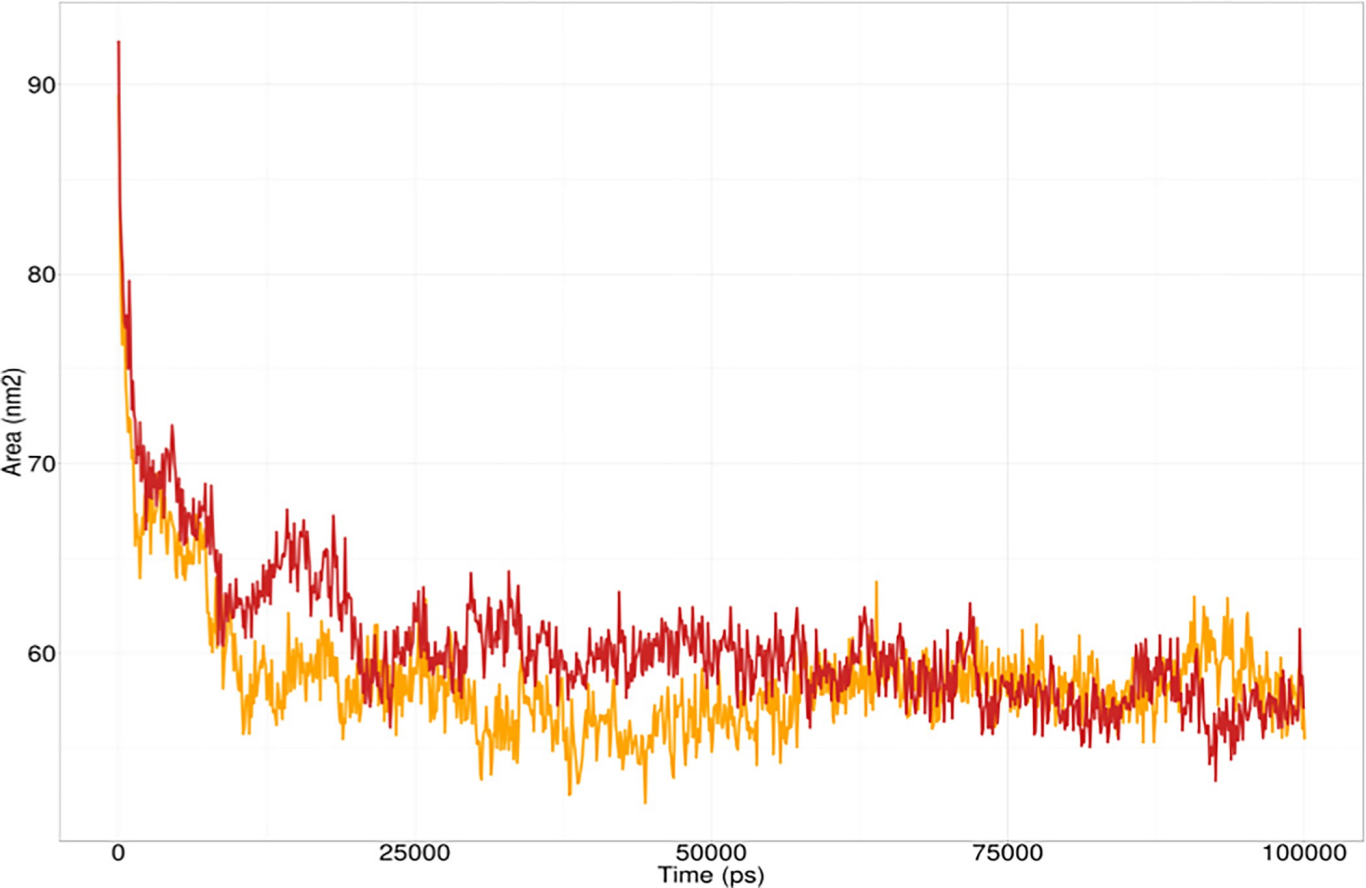
SASA analysis of wild type GRIN2A (Yellow) and I463S mutant GRIN2A protein (Red).

Solvent Accessible Surface Area (SASA) is used in MD simulations to predict the hydrophobic core stability of proteins. The higher the SASA value, the higher the chance of destabilization of the protein due to solvent accessibility. SASA values for the wild type and the I463S mutant *GRIN2A* remained close. However, the wild type displayed a slightly lower SASA profile compared to the other **(Fig 8**).

## Discussion

Almost half of the genetic variations that cause hereditary diseases come from nsSNPs [39]. Thorough research about the effect of these nsSNPs on disease-associated proteins can aid in the creation of more tailored, personalized treatments for the affected ones [40,41]. Although it can be laborious to identify which nsSNPs are responsible for specific symptoms due to the tedious and expensive nature of molecular approaches, bioinformatics can be used to predict which nsSNPs are pathogenic and prioritize them for future investigation [42,43]. This can help improve our understanding of the structure and function of proteins involved in these diseases.

Among the four genes (GRIN1, *GRIN2A*, GRIN2B, and GRIN2D) that encode NMDAR subunits that have so far been linked to human disease, *GRIN2A* appears to have the most extensive and well-characterized range of phenotypic effects [44]. Functional investigations of disease-associated *GRIN2A* missense variants have revealed various gain- or loss-of-function effects [45]. In a study by Endele et al. (2010), a de novo *GRIN2A* variant was found in a child with early-onset epileptic encephalopathy [46]. Another study by Gao et al., identified a new missense mutation in the *GRIN2A* gene in a patient with childhood focal epilepsy and acquired epileptic aphasia. This particular mutation was found to lower NMDAR activation implying that the decreased function of NMDAR could potentially be involved in the development of epilepsy [47]. Therefore, a detailed analysis of *GRIN2A* related nsSNPs is essential before starting targeted treatment options. In this study, we conducted intensive *in silico* analyses to identify the pathogenic nsSNPs of the *GRIN2A* gene. For this purpose, a wide range of computational tools was used to get a detailed understanding of the potential impact of these SNPs on the target gene.

To conduct our study, we retrieved all the available nsSNPs of the *GRIN2A* gene and annotated them using multiple computational tools with an aim to distinguish between the functional and neutral variants. The combination of these tools allowed us to produce a cohesive image of the potential pathogenic SNPs of the *GRIN2A* gene. To identify the high-risk SNPs, we isolated 16 nsSNPs that were predicted to be deleterious by all of the prediction algorithms, as these were considered to have a greater chance of being pathogenic.

The function, activity, and regulation of a protein are closely tied to its structural stability. When stability decreases, proteins undergo degradation, misfolding, and clumping, leading to eventual dysfunction [48]. To evaluate the impact of the 16 harmful nsSNPs on the stability of the GRIN2A protein, the nsSNPs were first analyzed using 3D structures of the mutated domains. Homology modeling was performed in order to obtain the 3D structure of the human GRIN2A protein due to the lack of existing X-ray crystallographic or NMR structure. Additionally, to determine the deleteriousness of the functional nsSNPs, the nsSNPs located in the conserved domains of the proteins were selected, as nsSNPs in highly conserved regions are more likely to cause harm than those located in the variable regions [49]. After undergoing homology modeling, model verification, secondary structure analysis, and interatomic interaction prediction, the list of mutations was narrowed down to I463S which exhibited the highest destabilizing effect on the protein.

Moreover, 100 ns long molecular dynamics simulation of the wild-type GRIN2A and I463S mutant GRIN2A proteins revealed that the mutation imposed a destabilizing effect on the structure of the GRIN2A protein under physiological conditions. Due to such destabilizing effects, protein functioning is also likely to be interrupted.

This study has reduced our knowledge gap about the polymorphisms of the *GRIN2A* gene and brought to our attention the probable presence of similar effect-causing variants. Consequently, a broader scale of human in vivo molecular imaging studies may be necessary to pinpoint the most impactful variants. In general, this study highlights the importance of continued research in this field to facilitate the development of customized treatment options for individuals with abnormalities related to *GRIN2A*. Besides, the insights obtained from this study may aid in developing novel drug-targeting strategies and biomarkers for those affected by *GRIN2A*-linked disorders.

## Conclusion

The identification of a potential high-risk variant of the *GRIN2A* gene in our study paves the way for further investigations into the impact of nsSNPs on the function of GRIN2A protein and their correlation with disease. In vivo models, genome-wide association studies, and clinical inspections can provide valuable insights into the biological mechanisms underlying these disorders and aid in the development of improved diagnostic and therapeutic strategies.

## Supporting information

S1 Fig Ramachandran plot of wild and mutant GRIN2A protein derived from PROCHECK. The plot shows the distribution of the residues within the most favored, additional allowed, generously allowed, and disallowed regions.
(PNG)
