# Supplementary figures and images for: Impact of highly deleterious non-synonymous polymorphisms on GRIN2A protein’s structure and function
Source: PLoS One. 2023 Jun 15;18(6):e0286917. doi: 10.1371/journal.pone.0286917 (PMC10270607; doi:10.1371/journal.pone.0286917)

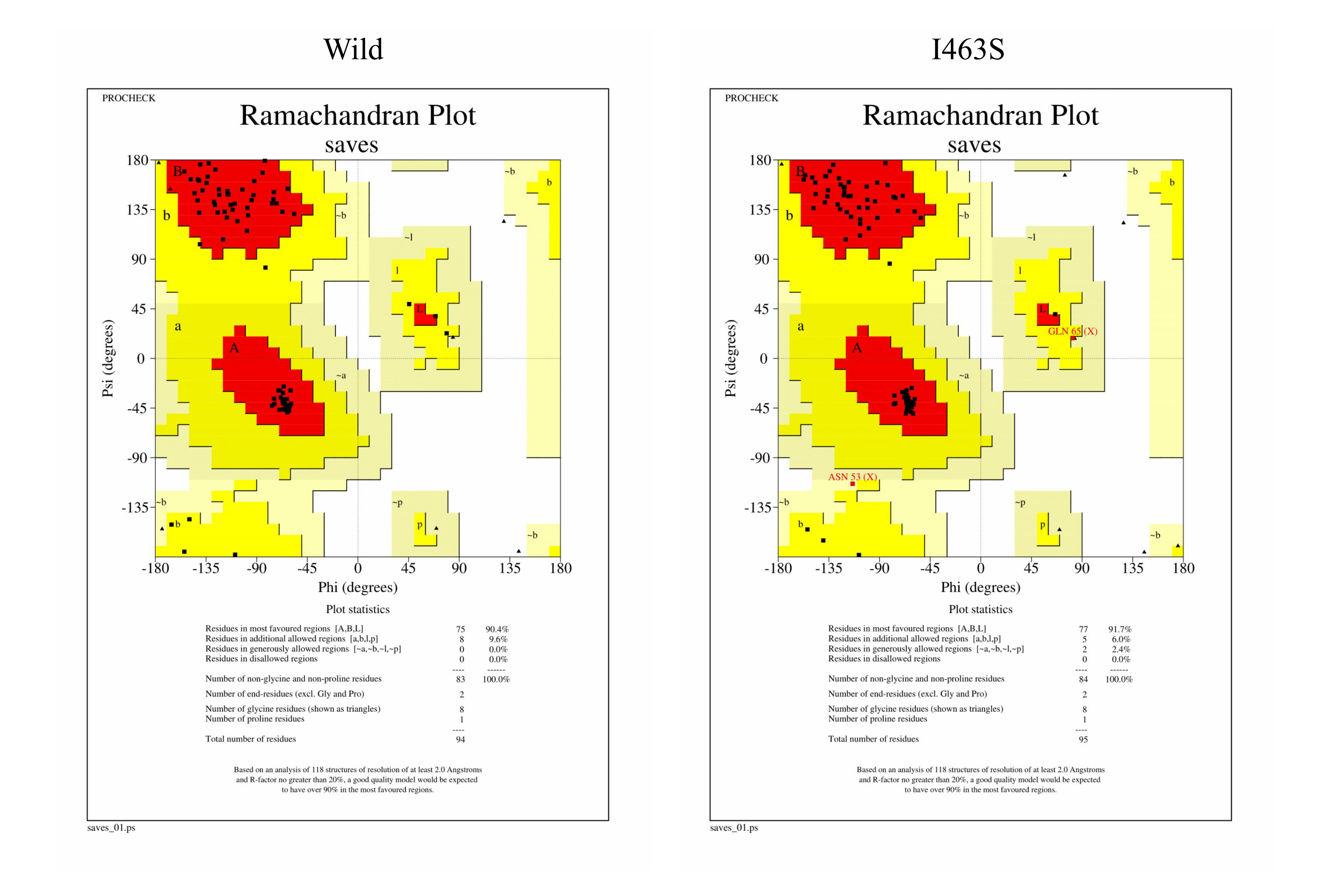

Supplement: S1 Fig — The plot shows the distribution of the residues within the most favored, additional allowed, generously allowed, and disallowed regions. (PNG) [file pone.0286917.s001.png]
